# Association of Sphingolipids with All-Cause and Cardiovascular Death in Patients with Kidney Failure Treated with Maintenance Hemodialysis

**DOI:** 10.1681/ASN.0000000982

**Published:** 2025-12-19

**Authors:** Benjamin Lidgard, Andrew N. Hoofnagle, Leila R. Zelnick, Ian H. de Boer, Paul Jensen, Amanda M. Fretts, David S. Siscovick, Jason G. Umans, Nisha Bansal, Rozenn N. Lemaitre

**Affiliations:** 1 Department of Medicine, University of Washington, Seattle, Washington; 2 New York Academy of Medicine, New York, New York; 3 Department of Medicine, Georgetown University, Washington, DC

**Keywords:** cardiovascular events, hemodialysis, lipids, mortality

## Abstract

**Key Points:**

- Sphingolipids with long fatty acids (16–18 carbons) were associated with all-cause and cardiovascular death in patients treated with hemodialysis.
- Sphingolipids with very-long fatty acids (20+ carbons) were associated with lower risk of all-cause and cardiovascular death in dialysis patients.

**Background:**

Patients with kidney failure on hemodialysis are at a higher risk for death, especially cardiovascular death. Statins (which improve outcomes in general populations) do not improve outcomes in dialysis patients. Sphingolipids are mechanistically associated with cardiovascular disease and may represent novel, modifiable risk factors for death in patients with kidney failure. We aimed to examine the associations of sphingolipids with death specifically in dialysis patients.

**Methods:**

Using data from the Hemodialysis (HEMO) Study (a multicenter factorial trial of dose and flux), we measured 16 sphingolipids (ceramides-16:0, 18:0, 20:0, 22:0, 24:0, and 24:1; hexosylceramides-16:0, 22:0, and 24:0; lactosylceramide-16:0; and sphingomyelins 14:0, 16:0, 18:0, 20:0, 22:0, and 24:0) at baseline only in 927 participants with available stored serum using targeted liquid chromatography–tandem mass spectrometry. The primary outcome was all-cause death, with physician-adjudicated cause (cardiovascular versus noncardiovascular) as a secondary outcome. We examined the associations of sphingolipids with death using Cox regressions, controlling the false discovery rate <5%.

**Results:**

Among 927 participants, the mean (SD) age was 57 (14) years; median (interquartile range) dialysis vintage was 1.9 (0.8–4.1) years. Over a median (interquartile range) follow-up of 2.4 (1.4–4.0) years, there were 376 deaths. Nine of 16 sphingolipids were significantly associated with death, including ceramide-16:0 (adjusted hazard ratio [aHR] 2.13 per two-fold higher concentration, 95% confidence interval, 1.48 to 3.06), and ceramide-22:0 (aHR per two-fold higher 0.59, 95% confidence interval, 0.44 to 0.79), with similar direction of associations for sphingomyelins. Twelve of 16 sphingolipids were associated with cardiovascular death, for example, ceramide-16:0 (aHR per two-fold higher 3.43, 95% confidence interval, 2.05 to 5.74). No sphingolipid was significantly associated with noncardiovascular death.

**Conclusions:**

In a dedicated study of patients with kidney failure on hemodialysis, sphingolipids with long-chain fatty acids were strongly associated with greater risk of death, especially cardiovascular death.

## Visual Abstract

**Figure:**
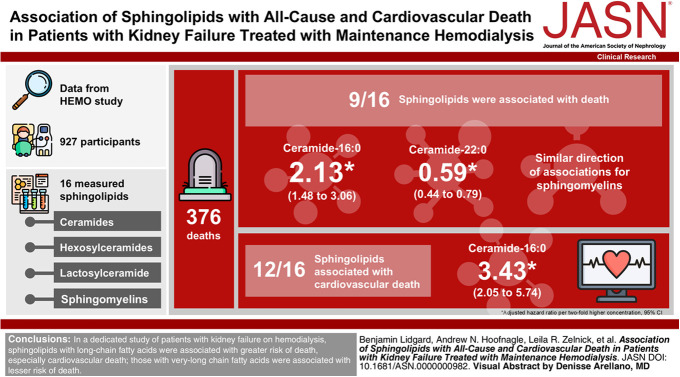

## Introduction

Patients with kidney failure treated with maintenance hemodialysis are at very high risk of death, especially from cardiovascular causes.^1–6^ Elevated low-density lipoprotein cholesterol is an important risk factor for death in the general population, although treatment of dialysis patients with statins has not been associated with improved outcomes.^7–10^ Lipid abnormalities other than low-density lipoprotein cholesterol may mediate the excess risk for death in dialysis patients.

Sphingolipids are a class of biologically active lipids with structural, regulatory, and signaling properties.^11–14^ Circulating concentrations of these lipids are associated with kidney function; lower eGFR and greater albuminuria have been associated with higher concentrations of long-chain sphingolipids and lower concentrations of very long–chain sphingolipids.^15–19^ Sphingolipids are also mechanistically implicated in the pathogenesis of cardiovascular disease and have been strongly associated with all-cause death in previous general population studies.^20–25^ The direction of these associations depends on the length of the acylated fatty acid; sphingolipids with long-chain fatty acids (14–18 carbons) are typically associated with greater risk of death, while those with very long–chain fatty acids (20+ carbons) are associated with lesser risk.^19,23,24,26–31^ Our group has shown that higher relative concentrations of sphingolipids with long-chain fatty acids in high-density lipoproteins (HDL) are associated with greater risk for death in patients with nondialysis requiring CKD; however, whether sphingolipids are also associated with all-cause and cardiovascular death in patients with kidney failure treated with maintenance hemodialysis is unknown.^19^ Identifying whether sphingolipids are associated with death in these patients may inform a novel therapeutic target in this high-risk population.

To investigate whether sphingolipids are associated with death in patients with kidney failure on maintenance hemodialysis, we leveraged data and samples from the Hemodialysis (HEMO) Study.^9,32,33^ We hypothesized that sphingolipids acylated to long-chain fatty acids would be associated with greater risk of all-cause and cardiovascular death and that sphingolipids acylated to very long–chain fatty acids would be associated with lesser risk.

## Methods

### Source Population

HEMO was a multicenter 2×2 factorial randomized clinical trial of dialysis dose and dialyzer flux among 1846 participants with kidney failure treated with maintenance hemodialysis. Between March 1995 and October 2000, participants from 15 centers in the Northeast, Southeast, Midwest, Southwest, and West regions of the United States were recruited and randomized to either standard-dose or high-dose dialysis and either a low-flux or high-flux dialyzer.^32^ Neither high-dose dialysis or high-flux dialyzer decreased overall mortality risk. All participants had commenced treatment with in-center hemodialysis at least 3 months before recruitment and had achieved a Kt/V >1.3 within 4.5 hours on two of three treatments during baseline. Individuals who were pregnant, scheduled for kidney transplant, or suffering from unstable angina or severe (New York Heart Association Class 4) heart failure were not eligible for the trial.

### Study Population

For the purposes of this study, we included all participants with an adequate volume of baseline serum available from the National Institute of Diabetes and Digestive and Kidney Diseases (NIDDK) repository (*n*=927). We did not apply additional inclusion or exclusion criteria.

### Sphingolipid Measurement

The primary exposures of interest were circulating concentrations of the following sphingolipids: ceramides 16:0, 18:0, 20:0, 22:0, 24:0, and 24:1; hexosylceramides 16:0, 22:0, and 24:0; lactosylceramide 16:0; and sphingomyelins 14:0, 16:0, 18:0, 20:0, 22:0, and 24:0. All sphingolipids were measured using baseline serum. The methods for evaluating these sphingolipids have been previously applied in multiple other cohorts and have been described in detail elsewhere.^17,22,34,35^ In brief, frozen serum was thawed, and sphingolipids were measured using liquid chromatography–tandem mass spectrometry with internal standards. All analyses assumed the presence of the most common d18:1 backbone, which is by far the most common in human plasma.^14,27,36,37^

We evaluated these sphingolipids individually (per two-fold higher concentration, or “doubling,” as in previous work) and by biologically plausible subgroups: ceramides with long acylated fatty acids (the sum of ceramides 16:0 and 18:0), sphingomyelins with long acylated fatty acids (the sum of sphingomyelins 14:0, 16:0, and 18:0), ceramides with very-long acylated fatty acids (the sum of ceramides 20:0, 22:0, 24:0, and 24:1), and sphingomyelins with very-long acylated fatty acids (the sum of sphingomyelins 20:0, 22:0, and 24:0).^17,22,23,38^

### Follow-Up and Determination of Death

The primary outcome of the HEMO Study was all-cause death, with cause of death (cardiovascular and noncardiovascular) constituting secondary outcomes.^32^ Follow-up on the primary outcome was censored at transition of dialysis modality, kidney transplant, withdrawal of consent, loss to follow-up, or end of administrative follow-up on December 31, 2001, whichever came first. Each death during follow-up was classified by the investigator at the Clinical Center and reported to the Data Coordinating Center.^33^ The data forming the basis for the reports were collected by study coordinators and included hospital records, International Classification of Diseases codes, clinic and dialysis unit notes, diagnostic tests (including electrocardiograms, echocardiograms, and other imaging studies), cardiac catheterization results, patient or family accounts, physician accounts, death certificates, and death notification forms to the United States Renal Data System. Death and cause of death were captured on all participants in HEMO by maintaining contact with dialysis units to which patients were transferred in case of transfer.

Cause of death was determined first by the Clinical Center Principal Investigator based on the above data; their comments and all other available information were then sent to the Outcome Review Committee for final determination of cause of death. All deaths were classified into one of 24 categories, four of which were deemed “cardiovascular death.” This included ischemic heart disease, congestive heart failure, arrhythmia or conduction problems, and “other” cardiovascular death, including myocarditis, endocarditis, pericarditis, and valvular heart disease. All other causes of death were deemed “noncardiovascular death.”

All death classifications by the Clinical Centers required independent audits by two members of the Outcome Review Committee assigned by the Data Coordinating Center. Agreement was required; if there was initial disagreement, the cause of death was adjudicated during a conference phone call of the entire Outcome Review Committee. In this study, we used all-cause death as our primary outcome, with cause of death (cardiovascular versus noncardiovascular death, as determined by the HEMO Study) serving as our secondary outcomes.

### Covariates

Sociodemographic factors included age at study enrollment, randomized intervention arms, self-reported race and ethnicity, and biologic sex. Dialysis factors included dialysis vintage, single-pool Kt/V at the time of sphingolipid measurement, and mean ultrafiltration rate in cc/kg per hour across all such available data for each participant; we did not include residual urine output given very high missingness in this covariate. Biometric factors included body mass index, total cholesterol, and albumin. Comorbidities included smoking (current, former, or never), alcohol use (current, former, or never), diabetes, heart failure, ischemic heart disease, stroke, and hypertension.

### Statistical Analyses

We tabulated baseline characteristics overall and by quartiles of ceramide 16:0, as we have used this sphingolipid in multiple previous studies.^17–19^ We evaluated the correlation between each sphingolipid with Pearson correlation coefficients. We calculated the unadjusted incidence rates (IR) of all-cause death overall and by quartiles of sphingolipids, generating 95% confidence intervals by bootstrapping with 2000 replicates.^39^ We examined the association between sphingolipid (per doubling in each sphingolipid) and all-cause death using Cox proportional hazards models. We applied a nested adjustment stratagem. Our first models were unadjusted. Adjusted model 1 included sociodemographic covariates. Adjusted model 2 additionally included dialysis characteristics, biometric and laboratory data, and comorbid conditions. Adjusted model 3 (our primary model) additionally adjusted each analysis for highly correlated sphingolipids with hypothesized opposed biologic effects. Sphingolipids with long acylated fatty acids were additionally adjusted for their 22:0 counterpart (for instance, ceramide 16:0 was adjusted for ceramide 22:0), and those with very-long acylated fatty acids were additionally adjusted for their corresponding 16:0 counterpart. This approach has been used by this group in several previous cohort studies; we calculated variance inflation factors for each model using the coadjustment scheme in this study.^17,19,22,23,34^ We prespecified to assess statistical significance using a Benjamini–Hochberg false discovery rate <0.05, given the hypothesis-generating nature of this study.^40–42^ We assessed for multiplicative interaction by age (< or ≥ the mean age of 56.5 years), race, biologic sex, known ischemic heart disease, and diabetes. All missing data were multiply imputed through generation of 20 complete datasets using the *mice* package. Missing covariates were multiply imputed using chained equations based on all other covariates present in our final models; standard diagnostics (trace plots, box and whisker plots, and density plots) were used to confirm the absence of trends and that imputed values in each dataset conformed to the expected ranges based on observed data. Estimates obtained using these imputed datasets were combined according to standard Rubin rules.^43–45^

In secondary analyses, we examined the association of each sphingolipid with the cause of death (cardiovascular versus noncardiovascular) as determined by the HEMO Study using cause-specific hazard ratios given their potential advantages for determining etiology of mortality. We used the same adjustment schemes as the primary analyses for these analyses.

As an additional secondary analysis attempting to simplify the data, we examined the associations of different sphingolipid subclasses with all-cause death (and causes of death) as above.^19,22,23,25^ For the purposes of these analyses, “long ceramides” were the sum of ceramide-16:0 and -18:0; “very long ceramides” were the sum of ceramide-20:0, -22:0, and 24:0; “long sphingomyelins” were the sum of sphingomyelin-14:0, -16:0, and -18:0; and “very long sphingomyelins” were the sum of sphingomyelin-20:0, -22:0, and -24:0. All sphingolipid subclasses were modeled per doubling. Finally, we examined the associations of the Ceramide Risk Score (using quartiles derived from this cohort, as this equation was originally developed in nondialysis requiring persons) with all-cause, cardiovascular, and noncardiovascular death.^34^

All analyses were completed in RStudio running *R* version 4.4.2 (*R* Foundation for Statistical Computing, Vienna, Austria).

## Results

### Characteristics of the Study Population

Among 927 participants, the mean (SD) age was 57 (14) years (Table 1). A total of 521 (56%) were women, and the majority reported a race of either Black (576, 62% of this study) or White (320, 35% of this study). The median (interquartile range [IQR]) dialysis vintage was 1.9 (0.8–4.1) years, and the mean (SD) averaged ultrafiltration rate was 12 (4) ml/kg per hour. In total, 360 (39%) had known heart failure, 418 (45%) had diabetes, and 743 (80%) used antihypertensive medications. Compared with participants with the lowest quartile of ceramide 16:0, those with the highest quartile of ceramide 16:0 tended to have a longer dialysis vintage, lower urine output, slightly higher total cholesterol, and higher prevalence of heart failure and diabetes. Characteristics of our study population were similar to those who were excluded from our present analyses given no available baseline serum (Supplemental Table 1).

**Table 1:** Characteristics of the study population receiving hemodialysis at baseline, overall, and by quartiles of ceramide-16:0

Characteristic	Overall	Quartile 1 (≤0.262 *µ*M)	Quartile 2 (0.262 to ≤0.315 *µ*M)	Quartile 3 (0.315 to ≤0.381 *µ*M	Quartile 4 (>0.381 *µ*M)
*N*	927	232	232	232	231
Demographics					
Age, mean (SD)	57 (14)	57 (14)	56 (15)	57 (14)	56 (14)
Women, *n* (%)	521 (56)	110 (47)	137 (59)	132 (57)	142 (61)
**Clinical site region, *n* (%)**					
Northeast	257 (28)	72 (31)	61 (26)	60 (26)	64 (28)
Southeast	254 (27)	67 (29)	65 (28)	69 (30)	53 (23)
Midwest	195 (21)	44 (19)	49 (21)	46 (20)	56 (24)
Southwest	71 (8)	11 (5)	14 (6)	23 (10)	23 (10)
West	150 (16)	38 (16)	43 (19)	34 (15)	35 (15)
**Race, *n* (%)**					
American Indian or Alaska Native	5 (1)	2 (1)	0 (0)	2 (1)	1 (0)
Asian, Pacific Islander, or Asian Indian	25 (3)	7 (3)	6 (3)	6 (3)	6 (3)
Black	576 (62)	142 (61)	149 (64)	146 (63)	139 (60)
Unknown	1 (0)	0 (0)	0 (0)	1 (0)	0 (0)
White	320 (35)	81 (35)	77 (33)	77 (33)	85 (37)
Hispanic ethnicity, *n* (%)	60 (6)	13 (6)	11 (5)	18 (8)	18 (8)
**Dialysis characteristics, mean (SD)**					
Dialysis vintage in yr, median (IQR)	1.9 (0.8–4.1)	1.6 (0.8–0.6)	1.9 (0.8–4.1)	1.9 (0.8–3.8)	2.4 (1.0–4.9)
Residual 24-h urine volume in ml, median (IQR)	150 (50–300)	168 (73–243)	145 (0–335)	175 (79–300)	125 (70–250)
Single-pool Kt/V	1.60 (0.27)	1.59 (0.30)	1.60 (0.26)	1.62 (0.27)	1.60 (0.26)
Ultrafiltration rate in ml/kg per hr	12 (4)	12 (3)	12 (3)	12 (3)	12 (4)
Blood flow rate	396 (55)	398 (55)	399 (54)	396 (58)	390 (54)
Dialysate flow rate	652 (137)	657 (136)	657 (138)	647 (137)	645 (137)
**Laboratory values, mean (SD)**					
Albumin, g/dl	4.0 (0.3)	3.9 (0.3)	3.9 (0.3)	3.9 (0.3)	3.8 (0.4)
Potassium, mEq/L	4.9 (0.8)	4.9 (0.8)	4.9 (0.7)	4.9 (0.8)	4.8 (0.8)
Total cholesterol, mg/dl	173 (40)	161 (35)	170 (35)	174 (36)	186 (47)
**Comorbidities, *N* (%)**					
Heart failure	360 (39)	83 (36)	87 (38)	94 (41)	96 (42)
Ischemic heart disease	346 (37)	91 (39)	73 (31)	89 (38)	93 (40)
Stroke	187 (20)	44 (19)	44 (19)	49 (21)	50 (22)
Diabetes mellitus	418 (45)	103 (44)	97 (42)	110 (47)	108 (47)
Predialysis systolic BP, mm Hg	152 (24)	151 (23)	149 (25)	155 (24)	151 (25)
Predialysis diastolic BP, mm Hg	82 (16)	82 (14)	81 (16)	84 (17)	82 (15)
Body mass index, kg/m^2^	26 (5)	26 (5)	26 (6)	25 (5)	25 (5)
Current smoking, *n* (%)	159 (17)	43 (19)	34 (15)	42 (18)	40 (17)
Current any alcohol use, *n* (%)	12 (1)	6 (3)	1 (0)	3 (1)	2 (1)
**Medication use, *N* (%)**					
Antihypertensives	743 (80)	186 (80)	191 (82)	185 (80)	181 (78)
Antihyperglycemics	289 (31)	63 (27)	71 (31)	76 (33)	79 (34)

IQR, interquartile range.

### Concentrations of Sphingolipids

Sphingomyelins were the most abundant serum sphingolipid in this study. For instance, the median (IQR) concentration of sphingomyelin-16:0 was 152 (131–174) *µ*M; by contrast, the median (IQR) concentration of ceramide-16:0 was 0.31 (0.26–0.38) *µ*M (Table 2). We observed rightward skew in most sphingolipids, which was normalized by applying a log-2 transformation.

**Table 2:** Distribution of sphingolipids in micromolar among participants in the Hemodialysis trial

Sphingolipid	Minimum	5th Percentile	25th Percentile	Median	75th Percentile	95th Percentile	Maximum
Ceramide-16:0	0.10	0.20	0.26	0.31	0.38	0.49	0.89
Ceramide-18:0	0.04	0.07	0.11	0.15	0.2	0.31	0.78
Ceramide-20:0	0.02	0.04	0.05	0.07	0.09	0.12	0.28
Ceramide-22:0	0.14	0.31	0.44	0.56	0.69	0.98	1.86
Ceramide-24:0	0.74	1.56	2.33	2.92	3.69	5.02	8.58
Ceramide-24:1	0.49	0.74	1.03	1.3	1.61	2.26	4.13
Hexosylceramide-16:0	0.04	0.08	0.12	0.15	0.19	0.26	0.5
Hexosylceramide-22:0	0.05	0.09	0.13	0.17	0.22	0.32	0.62
Hexosylceramide-24:0	0.04	0.09	0.14	0.18	0.23	0.32	0.56
Lactosylceramide-16:0	0.17	0.43	0.6	0.75	0.93	1.24	1.76
Sphingomyelin-14:0	4.22	16.85	25.8	33.4	42.2	58.1	96.5
Sphingomyelin-16:0	48.2	105.28	131	152	174	207	303
Sphingomyelin-18:0	13.8	22.7	31.3	38.6	47.6	64.5	99.4
Sphingomyelin-20:0	5.66	10.69	14.3	16.7	19.7	25.1	34.6
Sphingomyelin-22:0	8.34	16.06	21.5	25.8	31.4	41.3	59.8
Sphingomyelin-24:0	5.38	9.39	13.1	16.0	19.6	26.0	42.3

All sphingolipids were positively correlated with each other, with correlation coefficients ranging from 0.14 to 0.93 (Supplemental Figure 1). Particularly, strong correlation was noted within various subtypes of sphingolipids (for instance, hexosylceramides were strongly correlated with each other, but not with ceramides). In addition, sphingolipids (especially sphingomyelins) were strongly correlated across subtypes.

### Association of Sphingolipids with All-Cause Death

We observed a total of 376 total deaths over a median (IQR) follow-up of 2.4 (1.4–4.0) years. Of these, 186 (49%) were designated as cardiovascular deaths, and 190 were noncardiovascular deaths (Supplemental Figure 2). We observed higher unadjusted IR for death among participants with increasing quartiles of ceramides 16:0 and 18:0 and sphingomyelin 16:0 (Supplemental Table 2). For instance, participants with ceramide-16:0 in quartile 1 experienced 1.21 (95% confidence interval [CI], 0.95 to 1.47) deaths per 10 person-years, while those in quartile 4 experienced 1.82 (95% CI, 1.52 to 2.12) deaths per 10 person-years.

**Figure 1:**
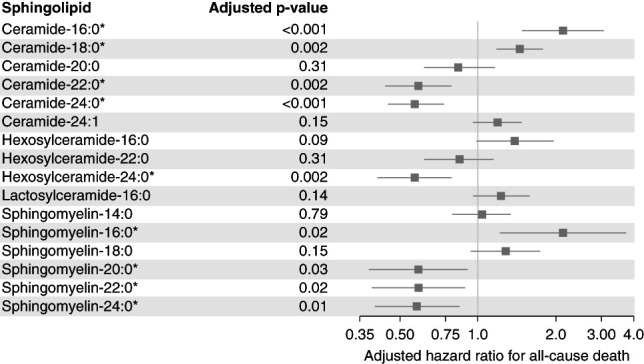
**Associations of sphingolipids with all-cause death.** aHR for all-cause death per two-fold higher concentration (* denotes statistical significance at an FDR <0.05). Adjusted for age, intervention arms, race, ethnicity, sex, BMI, cholesterol, albumin, ultrafiltration rate, Kt/V, dialysis vintage, predialysis systolic BP, ischemic heart disease, diabetes, stroke, and hypertension. In addition, sphingolipids with long-chain fatty acids were adjusted for the corresponding sphingolipid 22:0 (for instance, ceramide 16:0 was adjusted for ceramide 22:0), and sphingolipids with very long–chain fatty acids were adjusted for the corresponding sphingolipid 16:0 (for instance, sphingomyelin 24:0 was adjusted for sphingomyelin 16:0. aHR, adjusted hazard ratio; BMI, body mass index; FDR, false discovery rate.

In our primary analyses, each doubling of ceramide 16:0 was strongly associated with greater risk of death (adjusted cause-specific hazard ratio [adjusted hazard ratio (aHR) (95% CI) 2.13 (1.48 to 3.06)]; Figure 1 and Supplemental Table 3). Sphingomyelin 16:0 was strongly associated with all-cause death (aHR [95% CI] 2.13 [1.22 to 3.73]). Ceramide 18:0 was also associated with greater risk of all-cause death (aHR 1.45 [1.18 to 1.78]). Conversely, ceramides and sphingomyelins acylated to very long–chain fatty acids were associated with lesser risk for all-cause death. For instance, each doubling of ceramide 22:0 was associated with an aHR of 0.59 (95% CI, 0.44 to 0.79) for death; each doubling of sphingomyelin 22:0 was associated with an aHR of 0.59 (0.39 to 0.89) for death. Evaluation of Schoenfeld residuals did not demonstrate violation of the proportional hazards assumptions, evaluation of functional forms confirmed the linear assumption was appropriate (Supplemental Figure 3), and all variance inflation factors were <3.3 (Supplemental Table 4). We did not identify significant multiplicative interaction by age, race, sex, or history of ischemic heart disease or diabetes.

**Figure 2:**
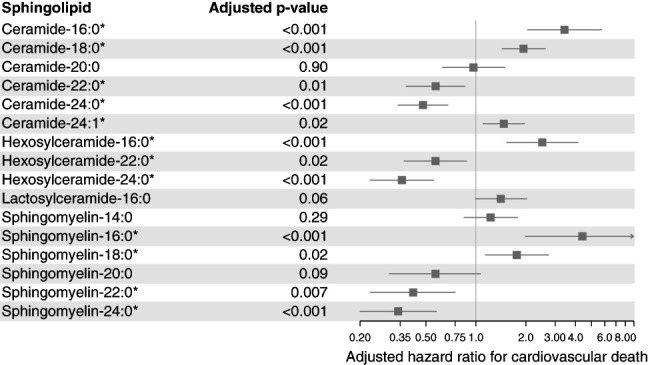
**Associations of sphingolipids with cardiovascular death.** aHR for cardiovascular death per two-fold higher concentration (* denotes statistical significance at an FDR <0.05). Adjusted for age, intervention arms, race, ethnicity, sex, BMI, cholesterol, albumin, ultrafiltration rate, Kt/V, dialysis vintage, predialysis systolic BP, ischemic heart disease, diabetes, stroke, and hypertension. In addition, sphingolipids with long-chain fatty acids were adjusted for the corresponding sphingolipid 22:0 (for instance, ceramide 16:0 was adjusted for ceramide 22:0), and sphingolipids with very long–chain fatty acids were adjusted for the corresponding sphingolipid 16:0 (for instance, sphingomyelin 24:0 was adjusted for sphingomyelin 16:0).

**Figure 3:**
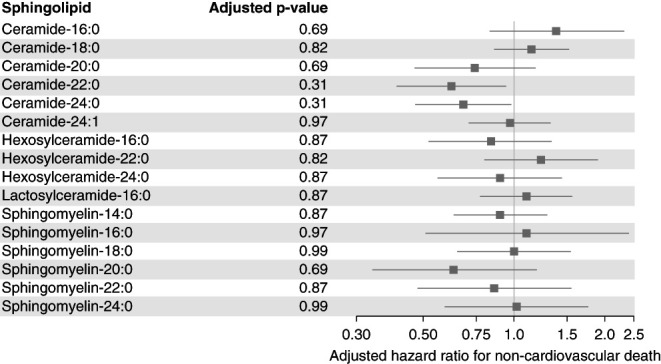
**Associations of sphingolipids with noncardiovascular death.** aHR for noncardiovascular death per two-fold higher concentration. Adjusted for age, intervention arms, race, ethnicity, sex, BMI, cholesterol, albumin, ultrafiltration rate, Kt/V, dialysis vintage, predialysis systolic BP, ischemic heart disease, diabetes, stroke, and hypertension. In addition, sphingolipids with long-chain fatty acids were adjusted for the corresponding sphingolipid 22:0 (for instance, ceramide 16:0 was adjusted for ceramide 22:0), and sphingolipids with very long–chain fatty acids were adjusted for the corresponding sphingolipid 16:0 (for instance, sphingomyelin 24:0 was adjusted for sphingomyelin 16:0).

In our first secondary analysis, we observed greater IRs for cardiovascular death across quartiles of ceramides 16:0, 18:0, and 20:0 and sphingomyelin 16:0 (Supplemental Table 5). For instance, participants with ceramide-16:0 in quartile 1 experienced 5.06 (95% CI, 3.33 to 6.79) cardiovascular deaths per 100 person-years, while the rate in quartile 4 was 9.34 (7.04 to 11.6) cardiovascular deaths per 100 person-years. Twelve of 16 sphingolipids were significantly associated with cardiovascular death at a false discovery rate <0.05. In particular, ceramide-16:0 and sphingomyelin-16:0 were associated with cardiovascular death (aHR [95% CI] for ceramide-16:0 3.43 [2.05 to 5.74]; for sphingomyelin-16:0 4.40 [1.98 to 9.82]; Figure 2 and Supplemental Table 6). Sphingolipids with very long–chain fatty acids were associated with lesser risk of this outcome; notably, each doubling in sphingomyelin-24:0 was associated with an aHR of 0.34 (95% CI, 0.20 to 0.58) for cardiovascular death. However, no sphingolipid was significantly associated with noncardiovascular death (Figure 3 and Supplemental Table 7).

**Figure 4:**
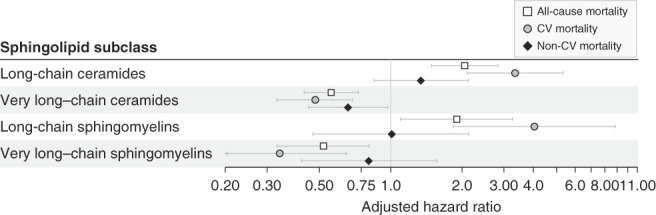
**Associations of sphingolipid subclasses with types of mortality.** aHR for each type of mortality per two-fold higher concentration. Adjusted for age, intervention arms, race, ethnicity, sex, BMI, cholesterol, albumin, ultrafiltration rate, Kt/V, dialysis vintage, predialysis systolic BP, ischemic heart disease, diabetes, stroke, and hypertension. In addition, sphingolipids with long-chain fatty acids were adjusted for the corresponding sphingolipid 22:0 (for instance, long-chain ceramides were adjusted for ceramide 22:0), and sphingolipids with very long–chain fatty acids were adjusted for the corresponding sphingolipid 16:0 (for instance, very long–chain sphingomyelins were adjusted for sphingomyelin 16:0). CV, cardiovascular.

In our additional secondary analysis, we modeled sphingolipids as posited subclasses. We observed greater unadjusted IR for death and cardiovascular death with higher quartiles of ceramides (IR [95% CI] in quartile 1: 1.13 [0.88 to 1.38] deaths per 10 person-years and 4.68 [3.01 to 6.35] cardiovascular deaths per 100 person-years versus quartile 4: 1.86 [1.55 to 2.18] deaths per 10 person-years and 10.9 [8.29 to 13.4] cardiovascular deaths per 100 person-years) and sphingomyelins acylated to long-chain fatty acids (Supplemental Tables 8 and 9). Each doubling in long-chain ceramides was associated with 2.05-fold (95% CI, 1.48 to 2.85) greater risk for all-cause death; each doubling in long-chain sphingomyelins was associated with 1.90-fold (1.10 to 3.26) greater risk for all-cause death (Figure 4 and Supplemental Table 10). Sphingolipids acylated to very long–chain fatty acids were associated with lesser risk for all-cause death (aHR for very long ceramides 0.56 [95% CI, 0.43 to 0.74]; aHR for very long sphingomyelins 0.52 [0.33 to 0.81]). Sphingolipids acylated to very long–chain fatty acids were associated with lesser risk for cardiovascular death (hazard ratio per two-fold higher sphingomyelins acylated to very-long fatty acids 0.34 [0.18 to 0.65]), while sphingolipids acylated to long-chain fatty acids were associated with greater risk of cardiovascular death (aHR per doubling of sphingomyelins acylated to very long–chain fatty acids 4.03 [1.84 to 8.85]; Figure 4 and Supplemental Table 10). No classes of sphingolipid were significantly associated with noncardiovascular death (Figure 4 and Supplemental Table 10). As with our primary analyses, we did not detect violation of the proportional hazards assumption, all variance inflation factor were <3.0 (Supplemental Table 11), and we did not detect multiplicative interaction. Finally, the Ceramide Risk Score was also associated with greater risk for death and cardiovascular death. Compared with participants with a score of 0–2, participants with a score of 10–12 had roughly two-fold greater risk for all-cause death and three-fold greater risk for cardiovascular death (Supplemental Table 12).

## Discussion

In this study, we demonstrated that sphingolipids with long-chain fatty acids were strongly associated with all-cause death (roughly two-fold greater risk for each two-fold higher sphingolipid concentration) in patients with kidney failure treated with maintenance hemodialysis, while higher concentrations of sphingolipids with very long–chain fatty acids were associated with lower risk of death. These associations were driven by the associations of sphingolipids with cardiovascular death, which were generally stronger in magnitude than those for all-cause mortality, and sphingolipids were not significantly associated with noncardiovascular death. This study may suggest the role of sphingolipids as a novel risk factor for cardiovascular death among such patients and potentially suggest novel therapeutic targets to reduce all-cause and cardiovascular death in this vulnerable population.

In this project, we described the distribution of sphingolipids in patients with kidney failure treated with maintenance hemodialysis; these concentrations may differ from the general population. For instance, we demonstrated a median ceramide-16:0 concentration of 0.31 *µ*M in dialysis patients. In patients with normal kidney function in the Cardiovascular Health Study, we previously reported a mean ceramide-16:0 concentration of 0.27 *µ*M; in younger patients in the Strong Heart Family study, the mean concentration was 0.20 *µ*M, lower than the levels observed in this study.^34,38^ Similarly, we noted higher concentrations of sphingomyelin-16:0 and lower concentrations of hexosylceramide-22:0 and -24:0 and sphingomyelin-18:0, -20:0, and -22:0 in dialysis patients compared with patients with normal kidney function.^34,38^ This is in line with previous studies demonstrating associations of lower eGFR with higher concentrations of sphingolipids with long-chain fatty acids and lower concentrations of sphingolipids with very long–chain fatty acids.^16,17,46^ The mechanisms by which kidney function may affect sphingolipid metabolism are not well understood, although it has been suggested that the kidneys may directly participate in *de novo* sphingolipid biosynthesis.^26^ For instance, mice with kidney injury accumulated higher concentrations of ceramide-16:0.^26^ Alternatively, kidney disease activates proinflammatory pathways that may upregulate *de novo* sphingolipid biosynthesis.^47–53^

We found that sphingolipids with long-chain fatty acids were associated with greater risk of death, especially cardiovascular death, while those with very long–chain fatty acids were associated with lesser risk of these outcomes. These associations have also been noted in patients with nondialysis requiring CKD and normal kidney function. For instance, in a smaller study of patients with CKD, we found that higher HDL abundance of ceramide-16:0 and sphingomyelin 16:0 were associated with greater risk of death.^19^ These lipids have also been associated with greater risk of death in multiple cohorts of free-living adults with normal kidney function, including the Strong Heart Family Study and the Cardiovascular Health Study.^34,54^ The strength of the associations between these sphingolipids and mortality appear similar to what was reported previously. For instance, we found each doubling of ceramide-16:0 and sphingomyelin-16:0 to be associated with a 2.13-fold greater mortality risk; each doubling in these lipids was associated with 1.89-fold and 2.51-fold, respectively, greater risk of mortality in the Cardiovascular Health Study. Each SD-greater concentration of these lipids was associated with 1.68-fold and 1.80-fold, respectively, greater risk for death in the Strong Heart Family Study. In addition, the associations of these sphingolipids, particularly ceramide-16:0, with major adverse cardiovascular events have been noted in a previous meta-analysis.^25^ Future work may be needed to explore if sphingolipids are associated with larger absolute risk differences for cardiovascular death in patients with kidney failure versus normal kidney function, given the much greater risk for cardiovascular risk in these patients.

The pathophysiology underlying sphingolipid-mediated death risk has not been well-described in general. However, it is known that sphingolipids with long-chain fatty acids are potent activators of proapoptotic pathways, and that they may increase risk for death through apoptosis leading to organ dysfunction.^48–50^ Similarly, basic science data support the role of sphingolipids in the pathogenesis of cardiovascular disease and the potential cardiotoxicity of these lipids. For instance, murine models developed increased intracardiomyocyte concentrations of ceramide-16:0, which led directly to the development of dilated cardiomyopathy and death.^51,52^ However, why sphingolipids with very long–chain fatty acids are associated with lesser risk of death is not known. As these sphingolipids are present in lipid rafts throughout the phospholipid bilayer, it has been hypothesized that they may contribute to membrane stability (sphingomyelins) and potentially decrease apoptotic signaling by competing with long-chain sphingolipids (ceramides).^11,36^ Further work is needed to understand the biologic mechanisms underlying these complex associations.

Mechanisms by which we may intervene on sphingolipid concentrations are currently being investigated. Sphingolipids are positively correlated because of the rate-controlling step of substrate synthesis.^12,31,49^ The activity of ceramide synthases (CerS), which acylate various fatty acids to sphinganine and sphingosine (in the *de novo* and salvage pathways, respectively), determines the length of the acylated fatty acid chain, and thus the associations with various outcomes.^12,31,53,55,56^ For instance, CerS2 primarily synthesizes very long–chain ceramides, while CerS 5–6 primarily synthesize ceramide-16:0. After the action of CerS, it does not seem possible to exchange the acylated chain. Therefore, there is interest in various therapies that may alter the expression of the various types of CerS; this work is ongoing. Less targeted methods focus on transport of sphingolipids in lipoproteins. For instance, as sphingolipids are present on the surface of low-density lipoprotein, it is not surprising that treatment with PCSK9 inhibitors and statins have been associated with lower circulating concentrations of total sphingolipids, especially those with long-chain fatty acids.^57^ While the effect of these medications are unknown in patients with nondialysis requiring CKD, let alone kidney failure, we have previously demonstrated that statins were not associated with HDL concentrations of ceramides or sphingomyelins, in cross-sectional observational analyses of people with CKD.^18^ Further work is needed to investigate how various therapies may alter circulating sphingolipid concentrations in other lipoproteins and specifically in patients with kidney disease. Similarly, while our results suggest that sphingolipids may be associated with adverse outcomes in patients with kidney failure, several considerations make their use as real-world biomarkers difficult. These include the specialized assays and equipment required to measure sphingolipids reliably, the cost of commercial assays, and the variability of these measurements in persons with and without kidney disease.

This study has several strengths. We used a dedicated trial of patients with kidney failure treated with maintenance hemodialysis to investigate the association of sphingolipids with all-cause death specifically in patients with kidney failure. The primary outcome (all-cause death) and the cause of death were rigorously physician-adjudicated by the HEMO trial, allowing us to perform evaluations of the association of sphingolipids with various types of death. Similarly, we measured all sphingolipids using a well-described tandem–mass spectrometry approach with internal standards that we have applied in multiple previous studies, assuring excellent reliability of measurements. However, there are some limitations. The HEMO Study was performed in the 1990s; dialysis therapy and (to a greater extent) cardiovascular disease prevention have improved since that time. The effect of the use of high-flux membranes on sphingolipids are unknown, although the HEMO Study did not find this to reduce the risk of death. HEMO did not record data on statin use; the effect of statin therapy on circulating sphingolipids is not well-known in persons with CKD (our group has noted no significant associations between statin use and HDL concentrations of sphingolipids in persons with nondialysis requiring CKD),^18^ and these medications do not decrease risk of death in persons treated with hemodialysis.^7,8,10^ Similarly, the fasting status of patients in the HEMO Study was not recorded, although the effect of diet on sphingolipid levels is not well-described. The median dialysis vintage was nearly 2 years, and the study population was well-treated; therefore, the results may not be generalizable to other patients. Residual kidney function was unknown in roughly 2/3 of participants in HEMO, which may affect sphingolipid concentrations and risk for mortality; however, we were unable to adjust for this covariate given the high degree of missingness. Consequently, our results may be confounded in part by residual kidney function. As with other epidemiologic studies of this type, sphingolipids were measured only at a single time point, at baseline, which likely oversimplifies complex relationships between sphingolipid metabolism and disease risk. Further work may be indicated in which sphingolipids are measured at multiple time points throughout the day. Finally, the study was composed of young, relatively healthy research volunteers who consented to a randomized trial, and we were unable to measure sphingolipids in all participants, as many did not have existing baseline serum samples at the time of our request to the NIDDK repository. These factors may all limit generalizability to other populations.

In conclusion, in a well-characterized study of patients with kidney failure treated with maintenance hemodialysis, we demonstrated that sphingolipids with long-chain fatty acids are strongly associated with greater risk of all-cause death, specifically from cardiovascular causes. Sphingolipids with very long–chain fatty acids were associated with lesser risk of these outcomes. Further work is required to investigate these findings in other populations with kidney disease and investigate how we may intervene on sphingolipid concentrations as a potential novel therapeutic approach.

## Supplementary Material

## Data Availability

Original data generated for the study will be made available upon reasonable request to the corresponding author. Data Type: Statistical Analysis Plan; sphingolipid measurements. Reason for Restricted Access: Access to the baseline HEMO Study data is controlled by the NIDDK. Data from the HEMO Study ([Version 5]) https://doi.org/10.58020/fmgh-4d37 reported here are available for request at the NIDDK Central Repository (NIDDK-CR) website, Resources for Research, https://repository.niddk.nih.gov.

## Supplemental Material

Supplemental Table 1. Characteristics of the HEMO Study at baseline, stratified by availability of baseline serum for the current study.

Supplemental Table 2. Unadjusted IR (bootstrapped 95% CI) for all-cause death per ten person-years by quartiles of sphingolipids.

Supplemental Table 3. Associations of sphingolipids with all-cause death expressed as hazard ratio (95% CI) per doubling in sphingolipid.

Supplemental Table 4. Variance inflation factors for sphingolipids by their coadjustment sphingolipid for each outcome.

Supplemental Table 5. IR (bootstrapped 95% CI) for cardiovascular death per 100 person-years by quartiles of sphingolipids.

Supplemental Table 6. Associations of sphingolipids with cardiovascular death expressed as hazard ratio (95% CI) per doubling in sphingolipid.

Supplemental Table 7. Associations of sphingolipids with noncardiovascular death expressed as hazard ratio (95% CI) per doubling in sphingolipid.

Supplemental Table 8. IR (95% CI) for all-cause death per ten person-years by quartiles of sphingolipid subclasses.

Supplemental Table 9. IR (95% CI) for cardiovascular death per 100 person-years by quartiles of sphingolipid subclasses.

Supplemental Table 10. Associations of sphingolipid subclasses with all-cause and cause-specific death expressed as hazard ratio (95% CI) per two-fold higher greater concentration of sphingolipid subclass.

Supplemental Table 11. Variance inflation factors for sphingolipid subclasses by their coadjustment sphingolipid for each outcome.

Supplemental Table 12. Outcomes among participants classified by Ceramide Risk Score.

Supplemental Figure 1. Correlation plot of measured sphingolipids at baseline.

Supplemental Figure 2. Causes of death in the cohort.

Supplemental Figure 3. Functional forms of the associations between circulating sphingolipids and all-cause death.
