# Supplementary material for: Association of Sphingolipids with All-Cause and Cardiovascular Death in Patients with Kidney Failure Treated with Maintenance Hemodialysis
Source: J Am Soc Nephrol. 2025 Dec 19;37(6):1237–47. doi: 10.1681/ASN.0000000982 (PMC13021012; doi:10.1681/ASN.0000000982)
Supplement: Supplementary file 1 [file jasn-37-1237-s001.pdf]

ASN American Society of Nephrology | JASN Journal of the American Society of Nephrology | KIDNEY360 Accessing Our World From Every Angle | CJASN Clinical Journal of the American Society of Nephrology

ASN Journal Disclosure Form

As per ASN journal policy, I have disclosed any financial relationships or commitments I have held in the past 36 months as included below. I have listed my Current Employer below to indicate there is a relationship requiring disclosure. If no relationship exists, my Current Employer is not listed.

N. Bansal reports the following:
Employer: University of Washington; Consultancy: AstraZeneca; Patents or Royalties: UptoDate; and Advisory or Leadership Role: Kidney360 Associate Editor; American College of Physicians.

I understand that the information above will be published within the journal article, if accepted, and that failure to comply and/or to accurately and completely report the potential financial conflicts of interest could lead to the following: 1) Prior to publication, article rejection, or 2) Post-publication, sanctions ranging from, but not limited to, issuing a correction, reporting the inaccurate information to the authors' institution, banning authors from submitting work to ASN journals for varying lengths of time, and/or retraction of the published work.

Name: Nisha Bansal
Manuscript ID: JASN-2025-001087R1
Manuscript Title: Association of sphingolipids with all-cause and cardiovascular death in patients with kidney failure treated with maintenance hemodialysis
Date of Completion: October 13, 2025
Disclosure Updated Date: January 7, 2025

ASN American Society of Nephrology | JASN Journal of the American Society of Nephrology | KIDNEY360 Accessing Our World From Every Angle | CJASN Clinical Journal of the American Society of Nephrology

ASN Journal Disclosure Form

As per ASN journal policy, I have disclosed any financial relationships or commitments I have held in the past 36 months as included below. I have listed my Current Employer below to indicate there is a relationship requiring disclosure. If no relationship exists, my Current Employer is not listed.

I. de Boer reports the following:
Employer: University of Washington; Consultancy: Alnylam, Boehringer-Ingelheim, DexCom, Lexicon, Lilly, Mitre, Novo Nordisk, Roche; Research Funding: DexCom, Novo Nordisk; Honoraria: National Institutes of Health; and Advisory or Leadership Role: Deputy Editor, Clinical Journal of the American Society of Nephrology; Clinical Practice Guideline Co-Chair, Kidney Disease Improving Global Outcomes; Past Chair, American Heart Association Kidney in Heart Disease Science Committee.

I understand that the information above will be published within the journal article, if accepted, and that failure to comply and/or to accurately and completely report the potential financial conflicts of interest could lead to the following: 1) Prior to publication, article rejection, or 2) Post-publication, sanctions ranging from, but not limited to, issuing a correction, reporting the inaccurate information to the authors' institution, banning authors from submitting work to ASN journals for varying lengths of time, and/or retraction of the published work.

Name: Ian de Boer
Manuscript ID: JASN-2025-001087R1
Manuscript Title: Association of sphingolipids with all-cause and cardiovascular death in patients with kidney failure treated with maintenance hemodialysis
Date of Completion: October 13, 2025
Disclosure Updated Date: August 5, 2025

ASN
American Society of Nephrology
JASN
Journal of the American Society of Nephrology
KIDNEY360
Accessing Our World From Every Angle
CJASN
Clinical Journal of the American Society of Nephrology

ASN Journal Disclosure Form

As per ASN journal policy, I have disclosed any financial relationships or commitments I have held in the past 36 months as included below. I have listed my Current Employer below to indicate there is a relationship requiring disclosure. If no relationship exists, my Current Employer is not listed.

A. Fretts reports the following:
Advisory or Leadership Role: National Institutes of Health, National Heart, Lung, and Blood Advisory Council member

I understand that the information above will be published within the journal article, if accepted, and that failure to comply and/or to accurately and completely report the potential financial conflicts of interest could lead to the following: 1) Prior to publication, article rejection, or 2) Post-publication, sanctions ranging from, but not limited to, issuing a correction, reporting the inaccurate information to the authors' institution, banning authors from submitting work to ASN journals for varying lengths of time, and/or retraction of the published work.

Name: Amanda M. Fretts
Manuscript ID: JASN-2025-001087R1
Manuscript Title: Association of sphingolipids with all-cause and cardiovascular death in patients with kidney failure treated with maintenance hemodialysis
Date of Completion: October 13, 2025
Disclosure Updated Date: June 10, 2025

ASN American Society of Nephrology
JASN Journal of the American Society of Nephrology
KIDNEY360 Accessing Our World From Every Angle
CJASN Clinical Journal of the American Society of Nephrology

ASN Journal Disclosure Form

As per ASN journal policy, I have disclosed any financial relationships or commitments I have held in the past 36 months as included below. I have listed my Current Employer below to indicate there is a relationship requiring disclosure. If no relationship exists, my Current Employer is not listed.

A. Hoofnagle reports the following:
Employer: University of Washington; Research Funding: Waters, Inc (a mass spectrometry company); and Advisory or Leadership Role: Clinical Chemistry (Associate Editor); College of American Pathologists (Chair, Accuracy-based Programs Committee).

I understand that the information above will be published within the journal article, if accepted, and that failure to comply and/or to accurately and completely report the potential financial conflicts of interest could lead to the following: 1) Prior to publication, article rejection, or 2) Post-publication, sanctions ranging from, but not limited to, issuing a correction, reporting the inaccurate information to the authors' institution, banning authors from submitting work to ASN journals for varying lengths of time, and/or retraction of the published work.

Name: Andrew N. Hoofnagle
Manuscript ID: JASN-2025-001087R1
Manuscript Title: Association of sphingolipids with all-cause and cardiovascular death in patients with kidney failure treated with maintenance hemodialysis
Date of Completion: October 22, 2025
Disclosure Updated Date: October 22, 2025

ASN American Society of Nephrology | JASN Journal of the American Society of Nephrology | KIDNEY360 Accessing Our World From Every Angle | CJASN Clinical Journal of the American Society of Nephrology

ASN Journal Disclosure Form

As per ASN journal policy, I have disclosed any financial relationships or commitments I have held in the past 36 months as included below. I have listed my Current Employer below to indicate there is a relationship requiring disclosure. If no relationship exists, my Current Employer is not listed.

P. Jensen has nothing to disclose.

I understand that the information above will be published within the journal article, if accepted, and that failure to comply and/or to accurately and completely report the potential financial conflicts of interest could lead to the following: 1) Prior to publication, article rejection, or 2) Post-publication, sanctions ranging from, but not limited to, issuing a correction, reporting the inaccurate information to the authors' institution, banning authors from submitting work to ASN journals for varying lengths of time, and/or retraction of the published work.

Name: Paul N Jensen
Manuscript ID: JASN-2025-001087R1
Manuscript Title: Association of sphingolipids with all-cause and cardiovascular death in patients with kidney failure treated with maintenance hemodialysis
Date of Completion: October 22, 2025
Disclosure Updated Date: October 22, 2025

ASN Journal Disclosure Form

As per ASN journal policy, I have disclosed any financial relationships or commitments I have held in the past 36 months as included below. I have listed my Current Employer below to indicate there is a relationship requiring disclosure. If no relationship exists, my Current Employer is not listed.

R. Lemaitre reports the following:
Employer: University of Washington; and Research Funding: NIH.

I understand that the information above will be published within the journal article, if accepted, and that failure to comply and/or to accurately and completely report the potential financial conflicts of interest could lead to the following: 1) Prior to publication, article rejection, or 2) Post-publication, sanctions ranging from, but not limited to, issuing a correction, reporting the inaccurate information to the authors' institution, banning authors from submitting work to ASN journals for varying lengths of time, and/or retraction of the published work.

Name: Rozenn Lemaitre
Manuscript ID: JASN-2025-001087R1
Manuscript Title: Association of sphingolipids with all-cause and cardiovascular death in patients with kidney failure treated with maintenance hemodialysis
Date of Completion: October 13, 2025
Disclosure Updated Date: October 13, 2025

ASN
American Society of Nephrology
JASN
Journal of the American Society of Nephrology
KIDNEY360
Accessing Our World From Every Angle
CJASN
Clinical Journal of the American Society of Nephrology

ASN Journal Disclosure Form

As per ASN journal policy, I have disclosed any financial relationships or commitments I have held in the past 36 months as included below. I have listed my Current Employer below to indicate there is a relationship requiring disclosure. If no relationship exists, my Current Employer is not listed.

B. Lidgard reports the following:
Employer: University of Washington; and Consultancy: Guidepoint.

I understand that the information above will be published within the journal article, if accepted, and that failure to comply and/or to accurately and completely report the potential financial conflicts of interest could lead to the following: 1) Prior to publication, article rejection, or 2) Post-publication, sanctions ranging from, but not limited to, issuing a correction, reporting the inaccurate information to the authors' institution, banning authors from submitting work to ASN journals for varying lengths of time, and/or retraction of the published work.

Name: Benjamin Lidgard
Manuscript ID: JASN-2025-001087
Manuscript Title: Association of sphingolipids with all-cause and cardiovascular death in patients with kidney failure treated with maintenance hemodialysis
Date of Completion: October 13, 2025
Disclosure Updated Date: February 4, 2025

ASN
American Society of Nephrology
JASN
Journal of the American Society of Nephrology
KIDNEY360
Accessing Our World From Every Angle
CJASN
Clinical Journal of the American Society of Nephrology

ASN Journal Disclosure Form

As per ASN journal policy, I have disclosed any financial relationships or commitments I have held in the past 36 months as included below. I have listed my Current Employer below to indicate there is a relationship requiring disclosure. If no relationship exists, my Current Employer is not listed.

D. Siscovick has nothing to disclose.

I understand that the information above will be published within the journal article, if accepted, and that failure to comply and/or to accurately and completely report the potential financial conflicts of interest could lead to the following: 1) Prior to publication, article rejection, or 2) Post-publication, sanctions ranging from, but not limited to, issuing a correction, reporting the inaccurate information to the authors' institution, banning authors from submitting work to ASN journals for varying lengths of time, and/or retraction of the published work.

Name: David Siscovick
Manuscript ID: JASN-2025-001087R1
Manuscript Title: Association of sphingolipids with all cause and cvd death in patients with kidney failure treated with maintenance hemodialysis
Date of Completion: October 23, 2025
Disclosure Updated Date: July 17, 2025

ASN American Society of Nephrology | JASN Journal of the American Society of Nephrology | KIDNEY360 Accessing Our World From Every Angle | CJASN Clinical Journal of the American Society of Nephrology

ASN Journal Disclosure Form

As per ASN journal policy, I have disclosed any financial relationships or commitments I have held in the past 36 months as included below. I have listed my Current Employer below to indicate there is a relationship requiring disclosure. If no relationship exists, my Current Employer is not listed.

J. Umans reports the following:
Employer: Medstar Health Research Institute (non profit)

I understand that the information above will be published within the journal article, if accepted, and that failure to comply and/or to accurately and completely report the potential financial conflicts of interest could lead to the following: 1) Prior to publication, article rejection, or 2) Post-publication, sanctions ranging from, but not limited to, issuing a correction, reporting the inaccurate information to the authors' institution, banning authors from submitting work to ASN journals for varying lengths of time, and/or retraction of the published work.

Name: Jason G. Umans
Manuscript ID: JASN-2025-001087R1
Manuscript Title: Association of sphingolipids with all-cause and cardiovascular death in patients with kidney failure treated with maintenance hemodialysis
Date of Completion: October 22, 2025
Disclosure Updated Date: October 22, 2025

ASN American Society of Nephrology
JASN Journal of the American Society of Nephrology
KIDNEY360 Accessing Our World From Every Angle
CJASN Clinical Journal of the American Society of Nephrology

ASN Journal Disclosure Form

As per ASN journal policy, I have disclosed any financial relationships or commitments I have held in the past 36 months as included below. I have listed my Current Employer below to indicate there is a relationship requiring disclosure. If no relationship exists, my Current Employer is not listed.

L. Zelnick reports the following:
Employer: University of Washington; and Advisory or Leadership Role: Statistical Editor for the American Society of Nephrology Journal Portfolio.

I understand that the information above will be published within the journal article, if accepted, and that failure to comply and/or to accurately and completely report the potential financial conflicts of interest could lead to the following: 1) Prior to publication, article rejection, or 2) Post-publication, sanctions ranging from, but not limited to, issuing a correction, reporting the inaccurate information to the authors' institution, banning authors from submitting work to ASN journals for varying lengths of time, and/or retraction of the published work.

Name: Leila R. Zelnick
Manuscript ID: JASN-2025-001087R3
Manuscript Title: Association of Sphingolipids with All-Cause and Cardiovascular Death in Patients with Kidney Failure Treated with Maintenance Hemodialysis
Date of Completion: December 5, 2025
Disclosure Updated Date: December 5, 2025
