# Supplementary material for: Association of Sphingolipids with All-Cause and Cardiovascular Death in Patients with Kidney Failure Treated with Maintenance Hemodialysis
Source: J Am Soc Nephrol. 2025 Dec 19;37(6):1237–47. doi: 10.1681/ASN.0000000982 (PMC13021012; doi:10.1681/ASN.0000000982)

Supplemental Material: Association of sphingolipids with all-cause and cardiovascular death in patients with kidney failure treated with maintenance hemodialysis

Benjamin Lidgard MD, Andrew N. Hoofnagle MD PhD, Leila R. Zelnick PhD, Ian H. de Boer MD, Paul Jensen PhD, Amanda M. Fretts PhD, David S. Siscovick MD, Jason G. Umans MD, Nisha Bansal MD, Rozenn N. Lemaitre PhD

Table of Contents

Page 2: Supplemental Table 1: Characteristics of the Hemodialysis Study at baseline, stratified by availability of baseline serum for the current study

Page 3: Supplemental Table 2: Unadjusted incidence rate (bootstrapped 95% confidence interval) for all-cause death per 10 person-years by quartiles of sphingolipids

Page 4: Supplemental Table: Associations of sphingolipids with all-cause death expressed as hazard ratio (95% confidence interval) per doubling in sphingolipid

Page 5: Supplemental Table 4: Variance inflation factors for sphingolipids by their co-adjustment sphingolipid for each outcome

Page 6: Supplemental Table 5: Incidence rate (bootstrapped 95% confidence interval) for cardiovascular death per 100 person-years by quartiles of sphingolipids

Page 7: Supplemental Table 6: Associations of sphingolipids with cardiovascular death expressed as hazard ratio (95% confidence interval) per doubling in sphingolipid

Page 8: Supplemental Table 7: Associations of sphingolipids with non-cardiovascular death expressed as hazard ratio (95% confidence interval) per doubling in sphingolipid

Page 9: Supplemental Table 8: Incidence rate (95% confidence interval) for all-cause death per 10 person-years by quartiles of sphingolipid subclasses

Page 10: Supplemental Table 9: Incidence rate (95% confidence interval) for cardiovascular death per 100 person-years by quartiles of sphingolipid subclasses

Page 11: Supplemental Table 10: Associations of sphingolipid sphingolipid subclasses with all-cause and cause-specific death expressed as hazard ratio (95% confidence interval) per two-fold higher greater concentration of sphingolipid subclass

Page 12: Supplemental Table 11: Variance inflation factors for sphingolipid sub-classes by their co-adjustment sphingolipid for each outcome

Page 13: Supplemental Table 12: Outcomes among participants classified by Ceramide Risk Score

Page 14: Supplemental Figure 1: Correlation plot of measured sphingolipids at baseline

Page 15: Supplemental Figure 2: Causes of death in the cohort

Page 16: Supplemental Figure 3: Functional forms of the associations between circulating sphingolipids and all-cause death

Supplemental Table 1: Characteristics of the Hemodialysis Study at baseline, stratified by availability of baseline serum for the current study

	Serum available (included in study)	Serum not available (excluded from study)
N	927	919
Age, mean (SD)	56 (14)	58 (14)
Women, n (%)	521 (56)	517 (56)
Clinical site region, n (%)		
Northeast	257 (28)	257 (28)
Southeast	254 (27)	323 (35)
Midwest	195 (21)	170 (18)
Southwest	71 (8)	44 (5)
West	150 (16)	125 (14)
Race, n (%)		
American Indian or Alaska Native	5 (1)	6 (1)
Asian, Pacific Islander, or Asian Indian	25 (3)	29 (3)
Black	576 (62)	580 (63)
Unknown	1 (0)	0 (0)
White	320 (35)	304 (33)
Hispanic ethnicity, n (%)	60 (6)	48 (5)
Dialysis vintage in years, median (IQR)	1.9 (0.8, 4.1)	2.4 (1.1, 5.3)
Residual 24-hour urine volume in mL, median (IQR)	150 (50, 300)	135 (0, 298)
Single-pool Kt/V	1.60 (0.27)	1.58 (0.26)
Ultrafiltration rate in mL/kg/hr	12 (4)	12 (4)
Blood flow rate	396 (55)	399 (54)
Dialysate flow rate	652 (137)	643 (136)
Albumin, g/dL	4.0 (0.3)	399 (54)
Potassium, mEq/L	4.9 (0.8)	399 (54)
Total cholesterol, mg/dL	173 (40)	172 (42)
Heart failure	360 (39)	373 (41)
Ischemic heart disease	346 (37)	379 (41)
Stroke	187 (20)	173 (19)
Diabetes mellitus	418 (45)	405 (44)
Pre-dialysis systolic blood pressure, mm Hg	152 (24)	152 (26)
Pre-dialysis diastolic blood pressure	82 (16)	81 (15)
Body mass index	26 (5)	25 (5)
Current smoking	159 (17)	161 (18)
Current any alcohol use	12 (1)	6 (1)
Antihypertensives	743 (80)	698 (76)
Antihyperglycemics	289 (31)	255 (28)

Supplemental Table 2: Unadjusted incidence rate (bootstrapped 95% confidence interval) for all-cause death per 10 person-years by quartiles of sphingolipids

Sphingolipid	Quartile 1	Quartile 2	Quartile 3	Quartile 4
Ceramide-16:0	1.21 (0.95, 1.47)	1.20 (0.95, 1.45)	1.63 (1.35, 1.91)	1.82 (1.52, 2.12)
Ceramide-18:0	1.18 (0.93, 1.43)	1.32 (1.06, 1.59)	1.54 (1.27, 1.82)	1.83 (1.52, 2.13)
Ceramide-20:0	1.28 (1.02, 1.54)	1.17 (0.92, 1.41)	1.73 (1.44, 2.02)	1.71 (1.41, 2.00)
Ceramide-22:0	1.60 (1.30, 1.90)	1.31 (1.04, 1.57)	1.49 (1.22, 1.75)	1.51 (1.24, 1.77)
Ceramide-24:0	1.84 (1.50, 2.17)	1.40 (1.13, 1.67)	1.33 (1.06, 1.60)	1.39 (1.15, 1.63)
Ceramide-24:1	1.27 (0.99, 1.54)	1.21 (0.96, 1.46)	1.71 (1.44, 1.97)	1.68 (1.39, 1.98)
Hexosylceramide-16:0	1.44 (1.16, 1.73)	1.29 (1.02, 1.55)	1.44 (1.17, 1.71)	1.72 (1.43, 2.00)
Hexosylceramide-22:0	1.46 (1.17, 1.74)	1.44 (1.16, 1.71)	1.48 (1.21, 1.75)	1.52 (1.24, 1.79)
Hexosylceramide-24:0	1.68 (1.36, 2.00)	1.41 (1.16, 1.67)	1.36 (1.09, 1.64)	1.47 (1.21, 1.73)
Lactosylceramide-16:0	1.34 (1.05, 1.64)	1.43 (1.15, 1.70)	1.43 (1.17, 1.68)	1.68 (1.40, 1.95)
Sphingomyelin-14:0	1.44 (1.15, 1.72)	1.30 (1.05, 1.55)	1.46 (1.18, 1.74)	1.70 (1.41, 1.99)
Sphingomyelin-16:0	1.34 (1.05, 1.63)	1.44 (1.18, 1.70)	1.50 (1.23, 1.76)	1.59 (1.32, 1.87)
Sphingomyelin-18:0	1.45 (1.17, 1.73)	1.46 (1.18, 1.73)	1.49 (1.21, 1.77)	1.49 (1.22, 1.76)
Sphingomyelin-20:0	1.49 (1.21, 1.78)	1.65 (1.36, 1.94)	1.28 (1.01, 1.54)	1.49 (1.23, 1.75)
Sphingomyelin-22:0	1.81 (1.49, 2.14)	1.42 (1.15, 1.69)	1.41 (1.14, 1.69)	1.30 (1.06, 1.54)
Sphingomyelin-24:0	1.96 (1.62, 2.29)	1.45 (1.16, 1.73)	1.21 (0.97, 1.45)	1.38 (1.13, 1.63)

Supplemental Table 3: Associations of sphingolipids with all-cause death expressed as hazard ratio (95% confidence interval) per doubling in sphingolipid

Sphingolipid	Unadjusted	Model 1	Model 2	Model 3	Adjusted p-value
Ceramide-16:0	1.60 (1.23, 2.09)	1.70 (1.30, 2.23)	1.55 (1.17, 2.05)	2.13 (1.48, 3.06)	<0.001*
Ceramide-18:0	1.30 (1.11, 1.52)	1.27 (1.08, 1.50)	1.26 (1.07, 1.49)	1.45 (1.18, 1.78)	0.002*
Ceramide-20:0	1.31 (1.07, 1.60)	1.22 (0.99, 1.50)	1.23 (0.99, 1.52)	0.84 (0.62, 1.16)	0.31
Ceramide-22:0	0.88 (0.71, 1.08)	0.85 (0.69, 1.05)	0.94 (0.75, 1.18)	0.59 (0.44, 0.79)	0.002*
Ceramide-24:0	0.72 (0.59, 0.88)	0.69 (0.56, 0.84)	0.76 (0.61, 0.96)	0.57 (0.45, 0.74)	<0.001*
Ceramide-24:1	1.34 (0.59, 1.65)	1.20 (0.97, 1.49)	1.19 (0.96, 1.47)	1.19 (0.96, 1.47)	0.15
Hexosylceramide-16:0	1.16 (0.94, 1.42)	1.40 (1.13, 1.74)	1.30 (1.04, 1.63)	1.39 (0.99, 1.96)	0.09
Hexosylceramide-22:0	1.03 (0.85, 1.24)	1.20 (0.99, 1.45)	1.13 (0.92, 1.38)	0.85 (0.62, 1.15)	0.31
Hexosylceramide-24:0	0.87 (0.72, 1.05)	1.03 (0.84, 1.25)	0.98 (0.80, 1.21)	0.57 (0.41, 0.79)	0.002*
Lactosylceramide-16:0	1.20 (0.95, 1.53)	1.29 (1.01, 1.63)	1.23 (0.96, 1.58)	1.23 (0.96, 1.58)	0.14
Sphingomyelin-14:0	1.19 (0.94, 1.40)	0.95 (0.77, 1.17)	1.03 (0.81, 1.30)	1.04 (0.80, 1.34)	0.79
Sphingomyelin-16:0	1.19 (0.83, 1.70)	1.55 (1.04, 2.29)	1.51 (0.99, 2.30)	2.13 (1.22, 3.73)	0.02*
Sphingomyelin-18:0	1.03 (0.82, 1.30)	1.11 (0.87, 1.42)	1.17 (0.91, 1.51)	1.28 (0.94, 1.74)	0.15
Sphingomyelin-20:0	0.90 (0.68, 1.19)	0.83 (0.61, 1.11)	0.94 (0.68, 1.30)	0.59 (0.38, 0.91)	0.03*
Sphingomyelin-22:0	0.73 (0.57, 0.95)	0.82 (0.62, 1.08)	0.93 (0.69, 1.27)	0.59 (0.39, 0.89)	0.02*
Sphingomyelin-24:0	0.68 (0.54, 0.87)	0.82 (0.63, 1.05)	0.91 (0.69, 1.21)	0.58 (0.40, 0.85)	0.01*

* Indicates significance at a False Discovery Rate <0.05
All VIF <3.2
Model 1 adjusted for age, intervention arms, sex, race, and ethnicity
Model 2 adjusted for the components of Model 1, as well as smoking, body mass index, total cholesterol, albumin, mean ultrafiltration rate, Kt/V, dialysis vintage, pre-dialysis systolic blood pressure, diabetes, heart failure, ischemic heart disease, stroke, and hypertension
Model 3 adjusted for the components of Model 2. Additionally, sphingolipids with long fatty acids (14-18 carbons) were additionally adjusted for the corresponding 22-carbon sphingolipid, and those with very-long fatty acids (≥20 carbons) were adjusted for the corresponding 16-carbon sphingolipid

Supplemental Table 4: Variance inflation factors for sphingolipids by their co-adjustment sphingolipid for each outcome

Sphingolipid (co-adjustment sphingolipid)	All-cause death	Cardiovascular death	Non-cardiovascular death
Ceramide-16:0 (ceramide-22:0)	1.90	1.82	1.97
Ceramide-18:0 (ceramide-22:0)	1.76	1.79	1.75
Ceramide-20:0 (ceramide-16:0)	2.60	2.71	2.60
Ceramide-22:0 (ceramide-16:0)	1.98	2.05	1.96
Ceramide-24:0 (ceramide-16:0)	1.42	1.44	1.45
Hexosylceramide 16:0 (hexosylceramide 22:0)	2.61	2.56	2.69
Hexosylceramide 22:0 (hexosylceramide 16:0)	2.72	2.78	2.69
Hexosylceramide 24:0 (hexosylceramide 16:0)	3.29	2.78	2.69
Sphingomyelin 14:0 (sphingomyelin 22:0)	1.77	1.67	1.64
Sphingomyelin 16:0 (sphingomyelin 22:0)	2.49	2.55	2.45
Sphingomyelin 18:0 (sphingomyelin 22:0)	2.02	2.11	1.97
Sphingomyelin 20:0 (sphingomyelin 16:0)	2.47	2.52	2.51
Sphingomyelin 22:0 (sphingomyelin 16:0)	2.65	2.77	2.57
Sphingomyelin 24:0 (sphingomyelin 16:0)	2.69	2.89	2.61

Supplemental Table 5: Incidence rate (95% confidence interval) for cardiovascular death per 100 person-years by quartiles of sphingolipids

Sphingolipid	Quartile 1	Quartile 2	Quartile 3	Quartile 4
Ceramide-16:0	5.06 (3.33, 6.79)	5.47 (3.68, 7.26)	9.07 (6.86, 11.3)	9.34 (7.04, 11.6)
Ceramide-18:0	4.86 (3.17, 6.56)	6.33 (4.41, 8.26)	6.89 (4.98, 8.80)	11.0 (8.48, 13.5)
Ceramide-20:0	5.35 (3.57, 7.14)	5.61 (3.80, 7.41)	8.82 (6.57, 11.1)	9.32 (7.07, 11.6)
Ceramide-22:0	7.09 (4.97, 9.20)	6.09 (4.16, 8.01)	8.39 (6.28, 10.5)	7.62 (5.66, 9.58)
Ceramide-24:0	8.51 (6.12, 10.9)	6.94 (4.99, 8.89)	7.02 (4.98, 9.07)	6.96 (5.10, 8.81)
Ceramide-24:1	5.02 (3.23, 6.80)	6.08 (4.22, 7.94)	8.1 (6.08, 10.12)	9.78 (7.44, 12.1)
Hexosylceramide-16:0	5.37 (3.49, 7.25)	7.46 (5.37, 9.54)	7.15 (5.23, 9.07)	8.97 (6.73, 11.2)
Hexosylceramide-22:0	6.69 (4.57, 8.82)	7.35 (5.32, 9.39)	8.16 (6.02, 10.3)	6.95 (5.00, 8.89)
Hexosylceramide-24:0	8.35 (5.90, 10.8)	6.62 (4.75, 8.49)	7.50 (5.42, 9.58)	6.96 (5.03, 8.89)
Lactosylceramide-16:0	6.11 (4.06, 8.17)	6.48 (4.55, 8.40)	6.75 (4.91, 8.59)	9.69 (7.29, 12.1)
Sphingomyelin-14:0	7.22 (5.07, 9.37)	5.55 (3.83, 7.28)	6.45 (4.51, 8.39)	10.0 (7.66, 12.4)
Sphingomyelin-16:0	6.17 (4.14, 8.20)	6.33 (4.50, 8.15)	8.01 (5.92, 10.1)	8.52 (6.31, 10.7)
Sphingomyelin-18:0	6.84 (4.83, 8.85)	6.84 (4.84, 8.84)	7.40 (5.41, 9.40)	8.03 (5.90, 10.2)
Sphingomyelin-20:0	6.73 (4.68, 8.78)	8.35 (6.13, 10.6)	5.65 (3.83, 7.47)	8.47 (6.29, 10.6)
Sphingomyelin-22:0	8.38 (6.04, 10.7)	7.06 (5.06, 9.06)	7.49 (5.44, 9.53)	6.50 (4.63, 8.36)
Sphingomyelin-24:0	10.1 (7.53, 12.6)	6.71 (4.72, 8.71)	6.06 (4.29, 7.84)	6.90 (4.93, 8.86)

Supplemental Table 6: Associations of sphingolipids with cardiovascular death expressed as hazard ratio (95% confidence interval) per doubling in sphingolipid

Sphingolipid	Unadjusted	Model 1	Model 2	Model 3	Adjusted p-value
Ceramide-16:0	2.50 (1.71, 3.64)	2.62 (1.78, 3.84)	2.31 (1.55, 3.46)	3.43 (2.05, 5.74)	<0.001*
Ceramide-18:0	1.64 (1.31, 2.06)	1.65 (1.31, 2.09)	1.61 (1.27, 2.04)	1.94 (1.44, 2.62)	<0.001*
Ceramide-20:0	1.70 (1.28, 2.26)	1.61 (1.20, 2.16)	1.60 (1.19, 2.14)	0.97 (0.63, 1.50)	0.90
Ceramide-22:0	1.08 (0.80, 1.45)	1.02 (0.76, 1.38)	1.12 (0.81, 1.54)	0.57 (0.38, 0.86)	0.01*
Ceramide-24:0	0.76 (0.57, 1.01)	0.72 (0.54, 0.96)	0.76 (0.56, 1.05)	0.48 (0.34, 0.68)	<0.001*
Ceramide-24:1	1.72 (0.57, 2.31)	1.58 (1.17, 2.14)	1.48 (1.10, 1.98)	1.48 (1.10, 1.98)	0.02*
Hexosylceramide-16:0	1.40 (1.05, 1.88)	1.73 (1.27, 2.35)	1.57 (1.13, 2.17)	2.52 (1.54, 4.14)	<0.001*
Hexosylceramide-22:0	1.05 (0.80, 1.37)	1.22 (0.93, 1.61)	1.08 (0.81, 1.45)	0.57 (0.37, 0.88)	0.02*
Hexosylceramide-24:0	0.88 (0.67, 1.15)	1.04 (0.79, 1.38)	0.92 (0.68, 1.24)	0.36 (0.23, 0.56)	<0.001*
Lactosylceramide-16:0	1.47 (1.05, 2.06)	1.53 (1.09, 2.15)	1.42 (1.00, 2.03)	1.42 (1.00, 2.03)	0.06
Sphingomyelin-14:0	1.77 (1.05, 1.85)	1.14 (0.85, 1.53)	1.19 (0.84, 1.67)	1.23 (0.85, 1.79)	0.29
Sphingomyelin-16:0	1.77 (1.05, 2.98)	2.38 (1.36, 4.17)	2.22 (1.22, 4.01)	4.40 (1.98, 9.82)	<0.001*
Sphingomyelin-18:0	1.26 (0.91, 1.75)	1.39 (0.98, 1.97)	1.44 (1.01, 2.07)	1.77 (1.14, 2.76)	0.02*
Sphingomyelin-20:0	1.11 (0.74, 1.66)	1.00 (0.66, 1.51)	1.14 (0.72, 1.80)	0.57 (0.30, 1.07)	0.09
Sphingomyelin-22:0	0.84 (0.58, 1.21)	0.89 (0.61, 1.32)	0.94 (0.61, 1.46)	0.42 (0.23, 0.75)	0.007*
Sphingomyelin-24:0	0.69 (0.49, 0.97)	0.80 (0.56, 1.14)	0.82 (0.55, 1.21)	0.34 (0.20, 0.58)	<0.001*

* Indicates significance at a False Discovery Rate <0.05
All VIF <2.9
Model 1 adjusted for age, intervention arms, sex, race, and ethnicity
Model 2 adjusted for the components of Model 1, as well as smoking, body mass index, total cholesterol, albumin, mean ultrafiltration rate, Kt/V, dialysis vintage, pre-dialysis systolic blood pressure, diabetes, heart failure, ischemic heart disease, stroke, and hypertension
Model 3 adjusted for the components of Model 2. Additionally, sphingolipids with long fatty acids (14-18 carbons) were additionally adjusted for the corresponding 22-carbon sphingolipid, and those with very-long fatty acids (≥20 carbons) were adjusted for the corresponding 16-carbon sphingolipid

Supplemental Table 7: Associations of sphingolipids with non-cardiovascular death expressed as hazard ratio (95% confidence interval) per doubling in sphingolipid

Sphingolipid	Unadjusted	Model 1	Model 2	Model 3	Adjusted p-value
Ceramide-16:0	1.03 (0.71, 1.49)	1.11 (0.76, 1.62)	1.08 (0.72, 1.63)	1.38 (0.83, 2.32)	0.69
Ceramide-18:0	1.03 (0.82, 1.29)	0.99 (0.79, 1.25)	1.03 (0.81, 1.31)	1.14 (0.86, 1.52)	0.82
Ceramide-20:0	1.01 (0.76, 1.34)	0.92 (0.68, 1.24)	0.95 (0.70, 1.30)	0.74 (0.47, 1.18)	0.69
Ceramide-22:0	0.72 (0.53, 0.96)	0.71 (0.53, 0.96)	0.81 (0.58, 1.12)	0.62 (0.41, 0.94)	0.31
Ceramide-24:0	0.69 (0.52, 0.91)	0.66 (0.49, 0.88)	0.77 (0.55, 1.07)	0.68 (0.47, 0.98)	0.31
Ceramide-24:1	1.06 (0.52, 1.42)	0.91 (0.67, 1.23)	0.97 (0.71, 1.32)	0.97 (0.71, 1.32)	0.97
Hexosylceramide-16:0	0.96 (0.72, 1.28)	1.15 (0.85, 1.56)	1.11 (0.81, 1.52)	0.84 (0.52, 1.33)	0.87
Hexosylceramide-22:0	1.00 (0.77, 1.31)	1.18 (0.89, 1.55)	1.19 (0.89, 1.59)	1.23 (0.80, 1.90)	0.82
Hexosylceramide-24:0	0.87 (0.66, 1.13)	1.02 (0.77, 1.35)	1.05 (0.78, 1.41)	0.90 (0.56, 1.44)	0.87
Lactosylceramide-16:0	0.99 (0.71, 1.38)	1.09 (0.78, 1.52)	1.10 (0.77, 1.56)	1.10 (0.77, 1.56)	0.87
Sphingomyelin-14:0	0.81 (0.73, 1.26)	0.79 (0.59, 1.05)	0.92 (0.65, 1.28)	0.90 (0.63, 1.29)	0.87
Sphingomyelin-16:0	0.81 (0.49, 1.33)	1.00 (0.58, 1.73)	1.08 (0.59, 1.98)	1.10 (0.51, 2.40)	0.97
Sphingomyelin-18:0	0.85 (0.61, 1.17)	0.88 (0.62, 1.25)	1.01 (0.70, 1.46)	1.00 (0.65, 1.54)	0.99
Sphingomyelin-20:0	0.73 (0.49, 1.08)	0.68 (0.44, 1.04)	0.81 (0.51, 1.30)	0.63 (0.34, 1.19)	0.69
Sphingomyelin-22:0	0.64 (0.44, 0.92)	0.76 (0.51, 1.11)	0.97 (0.62, 1.50)	0.86 (0.48, 1.55)	0.87
Sphingomyelin-24:0	0.68 (0.48, 0.94)	0.84 (0.59, 1.21)	1.06 (0.70, 1.60)	1.02 (0.59, 1.76)	0.99

* Indicates significance at a False Discovery Rate <0.05
All VIF <3.0
Model 1 adjusted for age, intervention arms, sex, race, and ethnicity
Model 2 adjusted for the components of Model 1, as well as smoking, body mass index, total cholesterol, albumin, mean ultrafiltration rate, Kt/V, dialysis vintage, pre-dialysis systolic blood pressure, diabetes, heart failure, ischemic heart disease, stroke, and hypertension
Model 3 adjusted for the components of Model 2. Additionally, sphingolipids with long fatty acids (14-18 carbons) were additionally adjusted for the corresponding 22-carbon sphingolipid, and those with very-long fatty acids (≥20 carbons) were adjusted for the corresponding 16-carbon sphingolipid

Supplemental Table 8: Incidence rate (bootstrapped 95% confidence interval) for all-cause death per 10 person-years by quartiles of sphingolipid subclasses

Sphingolipid sub-class	Quartile 1	Quartile 2	Quartile 3	Quartile 4
Long-chain ceramides	1.13 (0.88, 1.38)	1.32 (1.06, 1.59)	1.55 (1.29, 1.82)	1.86 (1.55, 2.18)
Long-chain sphingomyelins	1.25 (0.98, 1.53)	1.45 (1.19, 1.71)	1.48 (1.21, 1.74)	1.68 (1.39, 1.97)
Very long-chain ceramides	1.83 (1.50, 2.16)	1.36 (1.10, 1.63)	1.34 (1.07, 1.61)	1.43 (1.18, 1.67)
Very long-chain sphingomyelins	1.72 (1.41, 2.03)	1.56 (1.26, 1.85)	1.21 (0.95, 1.47)	1.46 (1.21, 1.71)

Supplemental Table 9: Incidence rate (bootstrapped 95% confidence interval) for cardiovascular death per 100 person-years by quartiles of sphingolipid subclasses

Sphingolipid subclass	Quartile 1	Quartile 2	Quartile 3	Quartile 4
Long-chain ceramides	4.68 (3.01, 6.35)	5.53 (3.69, 7.38)	7.92 (5.89, 9.94)	10.9 (8.29, 13.4)
Long-chain sphingomyelins	5.58 (3.66, 7.51)	6.27 (4.43, 8.10)	7.47 (5.49, 9.44)	9.64 (7.28, 12.0)
Very long-chain ceramides	7.91 (5.57, 10.3)	7.06 (5.09, 9.09)	7.15 (5.12, 9.19)	7.21 (5.29, 9.12)
Very long-chain sphingomyelins	8.32 (6.04, 10.6)	7.67 (5.51, 9.82)	5.92 (4.11, 7.73)	7.51 (5.51, 9.52)

Supplemental Table 10: Associations of sphingolipid subclasses with all-cause and cause-specific death expressed as hazard ratio (95% confidence interval) per two-fold greater concentration of sphingolipid subclass

All-cause death	Unadjusted HR (95% CI)	Model 1 HR (95% CI)	Model 2 HR (95% CI)	Model 3 HR (95% CI)	Adjusted p-value
Long ceramides	1.54 (1.22, 1.94)	1.57 (1.24, 1.99)	1.48 (1.16, 1.90)	2.05 (1.48, 2.85)	<0.001*
Long sphingomyelins	1.20 (0.85, 1.70)	1.31 (0.91, 1.89)	1.40 (0.94, 2.08)	1.90 (1.10, 3.26)	0.02*
Very long ceramides	0.75 (0.61, 0.92)	0.71 (0.58, 0.88)	0.79 (0.63, 1.00)	0.56 (0.43, 0.74)	<0.001*
Very long sphingomyelins	0.73 (0.56, 0.96)	0.81 (0.60, 1.07)	0.92 (0.67, 1.27)	0.52 (0.33, 0.81)	0.006*
Cardiovascular death	Unadjusted HR (95% CI)	Model 1 HR (95% CI)	Model 2 HR (95% CI)	Model 3 HR (95% CI)	Adjusted p-value
Long ceramides	2.27 (1.64, 3.15)	2.34 (1.67, 3.28)	2.16 (1.52, 3.06)	3.34 (2.10, 5.32)	<0.001*
Long sphingomyelins	1.80 (1.10, 2.94)	1.98 (1.18, 3.33)	2.04 (1.16, 3.58)	4.03 (1.84, 8.85)	<0.001*
Very long ceramides	0.81 (0.61, 1.09)	0.77 (0.57, 1.03)	0.83 (0.60, 1.14)	0.48 (0.33, 0.69)	<0.001*
Very long sphingomyelins	0.83 (0.56, 1.21)	0.87 (0.58, 1.31)	0.94 (0.59, 1.48)	0.34 (0.18, 0.65)	0.001*
Non-cardiovascular death	Unadjusted HR (95% CI)	Model 1 HR (95% CI)	Model 2 HR (95% CI)	Model 3 HR (95% CI)	Adjusted p-value
Long ceramides	1.04 (0.74, 1.44)	1.05 (0.75, 1.48)	1.07 (0.75, 1.53)	1.34 (0.95, 2.13)	0.42
Long sphingomyelins	0.81 (0.51, 1.31)	0.86 (0.52, 1.44)	1.03 (0.58, 1.83)	1.01 (0.47, 2.14)	0.98
Very long ceramides	0.68 (0.51, 0.91)	0.66 (0.49, 0.89)	0.77 (0.55, 1.08)	0.66 (0.45, 0.97)	0.14
Very long sphingomyelins	0.64 (0.44, 0.95)	0.74 (0.49, 1.12)	0.95 (0.60, 1.52)	0.81 (0.42, 1.56)	0.53

* Indicates significance at a False Discovery Rate <0.05
Long ceramides were the sum of ceramide-16:0 and ceramide-18:0
Long sphingomyelins were the sum of sphingomyelin-14:0, -16:0, and -18:0
Very long ceramides were the sum of ceramide-20:0, -22:0, and -24:0
Very long sphingomyelins were the sum of sphingomyelin-20:0, -22:0, and -24:0
Model 1 adjusted for age, intervention arms, sex, race, and ethnicity
Model 2 adjusted for the components of Model 1, as well as smoking, body mass index, total cholesterol, albumin, mean ultrafiltration rate, Kt/V, dialysis vintage, pre-dialysis systolic blood pressure, diabetes, heart failure, ischemic heart disease, stroke, and hypertension
Model 3 adjusted for the components of Model 2. Additionally, sphingolipids with long fatty acids (14-18 carbons) were additionally adjusted for the corresponding 22-carbon sphingolipid, and those with very-long fatty acids (≥20 carbons) were adjusted for the corresponding 16-carbon sphingolipid

Supplemental Table 11: Variance inflation factors for sphingolipid sub-classes by their co-adjustment sphingolipid for each outcome

Sphingolipid (co-adjustment sphingolipid)	All-cause death	Cardiovascular death	Non-cardiovascular death
Long-chain ceramides (ceramide-22:0)	1.95	1.90	2.02
Long-chain sphingomyelins (sphingomyelin-22:0)	2.50	2.58	2.47
Very-long ceramides (ceramide-16:0)	1.50	1.56	1.54
Very-long sphingomyelins (sphingomyelin-16:0)	2.83	2.99	2.81

Supplemental Table 12: Outcomes among participants classified by Ceramide Risk Score

Ceramide Risk Score	0-2	3-6	7-9	10-12
Participants, n (%)	338	328	151	110
All-cause death, n (%)	97 (28.7)	134 (40.9)	78 (51.7)	67 (60.9)
CV death, n (%)	41 (12.1)	56 (17.1)	51 (33.8)	38 (34.5)
Non-CV death, n (%)	56 (16.6)	78 (23.8)	27 (17.9)	29 (26.4)
Adjusted hazard ratios (95% confidence intervals) for outcomes				
All-cause death	Ref	1.40 (1.08, 1.83)	1.73 (1.26, 2.36)	2.06 (1.49, 2.85)
CV death	Ref	1.36 (0.91, 2.05)	2.52 (1.64, 3.86)	2.94 (1.85, 4.66)
Non-CV death	Ref	1.51 (1.06, 2.14)	1.11 (0.69, 1.80)	1.55 (0.96, 2.49)

All models were adjusted for age, intervention arms, sex, race, ethnicity, smoking, body mass index, total cholesterol, albumin, mean ultrafiltration rate, Kt/V, dialysis vintage, pre-dialysis systolic blood pressure, diabetes, heart failure, ischemic heart disease, stroke, and hypertension.

Supplemental Figure 1: Correlation plot of measured sphingolipids at baseline

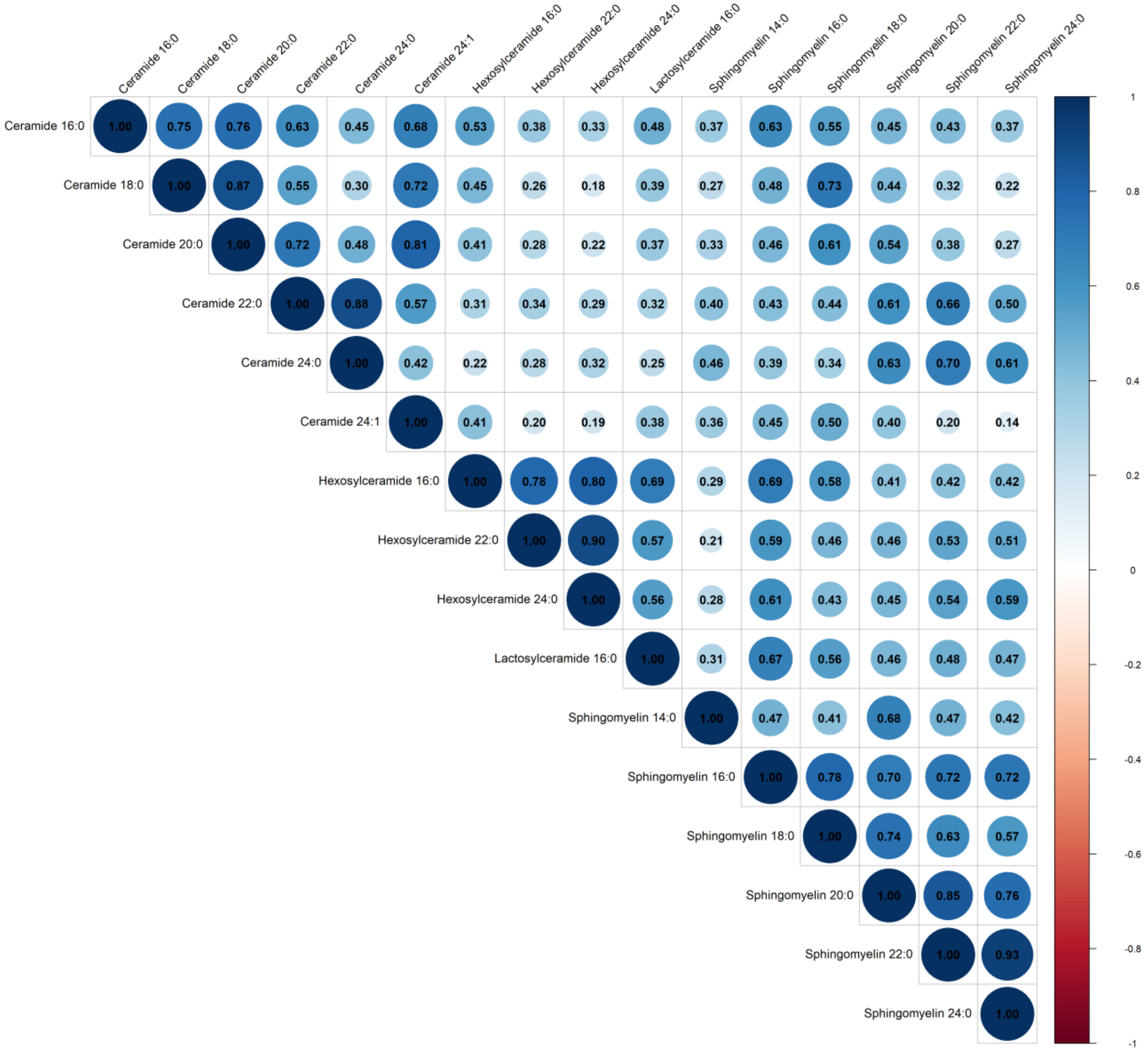

Supplemental Figure 2: Causes of death in the cohort

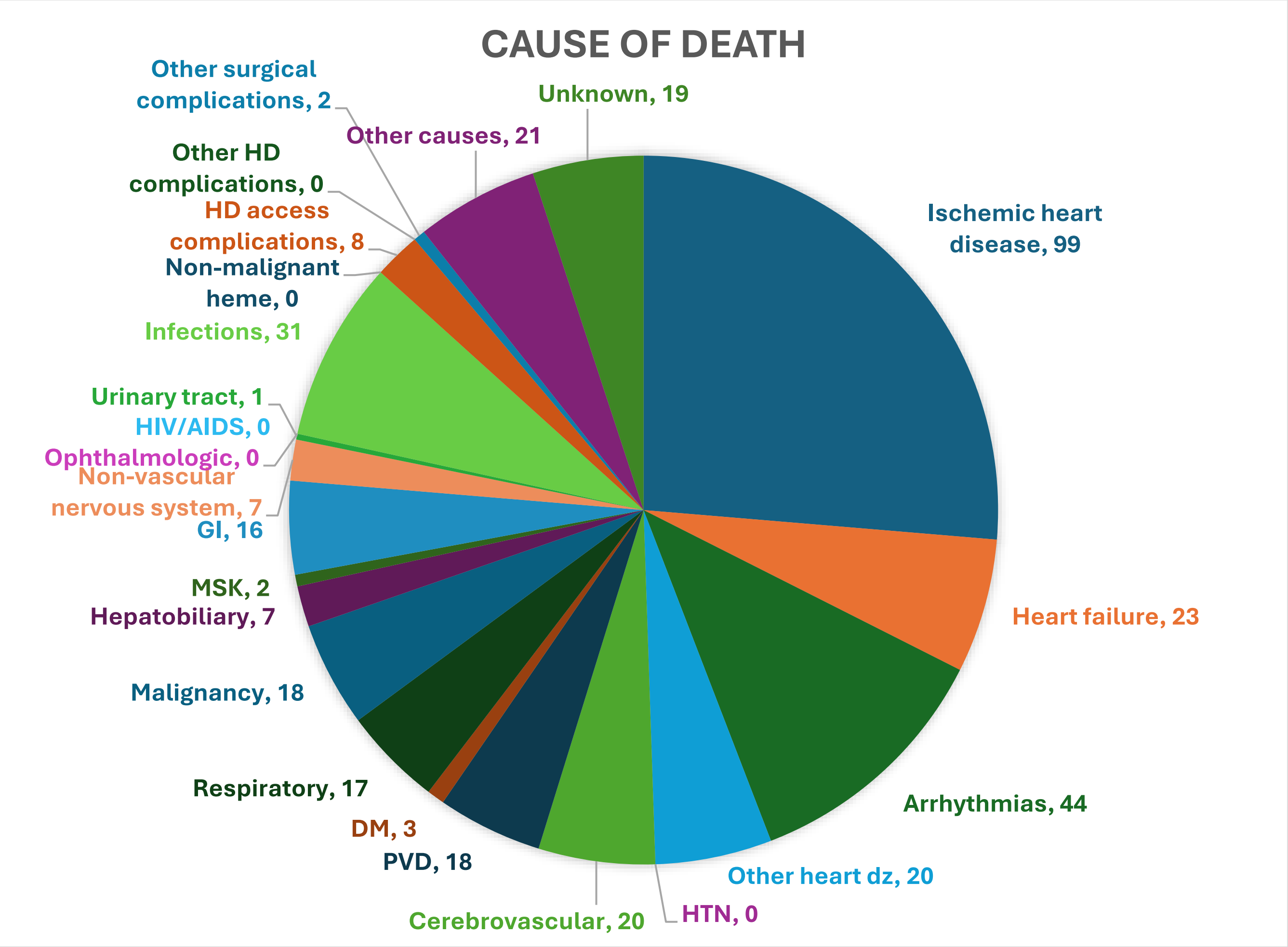

Supplemental Figure 3: Functional forms of the associations between circulating sphingolipids and all-cause death

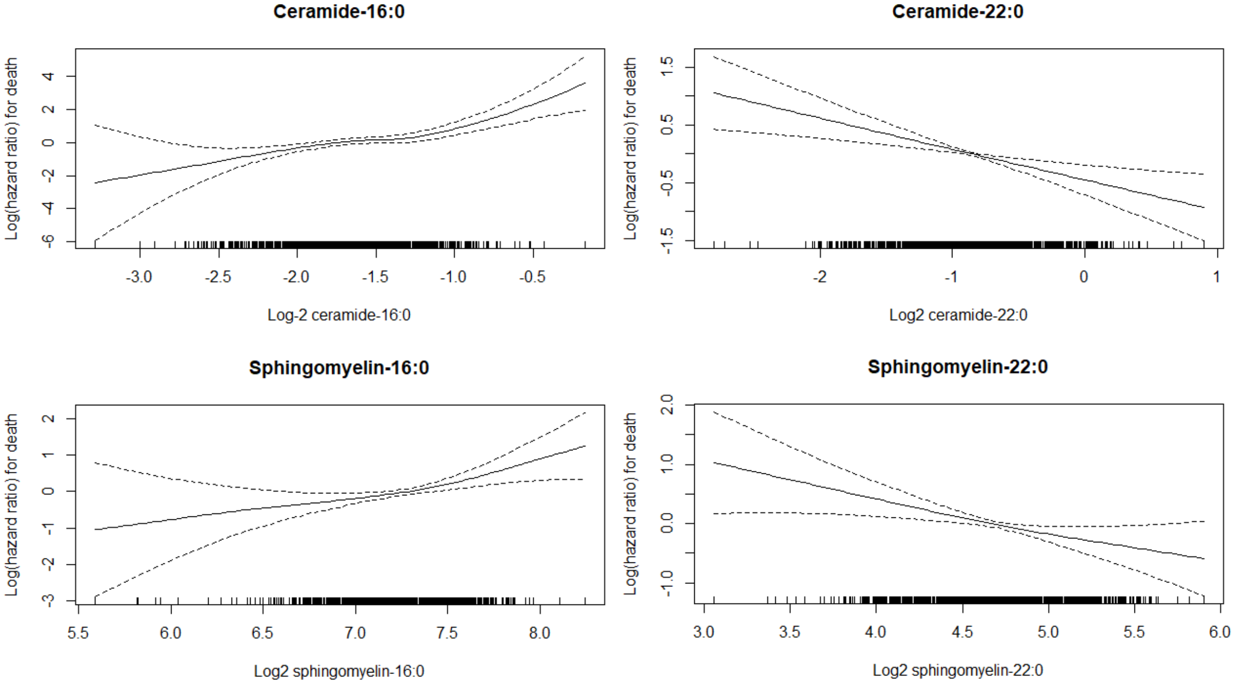

Supplement: Supplementary file 2 [file jasn-37-1237-s002.pdf]
